# Early Miscarriage Occurring Six Days After Intravitreal Ranibizumab Injection

**Published:** 2019

**Authors:** Sezen AKKAYA

**Affiliations:** Department of Ophthalmology, Fatih Sultan Mehmet Training and Research Hospital, İstanbul, Turkey

**Keywords:** Spontaneous Abortion, Intravitreal Ranibizumab, Diabetic Macular Edema, Pregnancy Tests

## Abstract

The aim of this case report was to describe a miscarriage which occurred 6 days after an intravitreal Ranibizumab (IVR) injection. A 24-year-old female patient with type 1 diabetes diagnosed with diabetic macular edema in her left eye planned for 3 injections of IVR at one-month intervals. She had been receiving insulin injections 3 times a day and her Hemoglobin A1C (HbA1c) was in the approximate range of 6–7%. An ophthalmologic examination revealed that the patient’s Snellen corrected distance visual acuity (CDVA) was 10/10 in her right eye and 3/10 in her left eye. The patient was unaware of her pregnancy at the time of initial injection. Two days after the first injection, she found out that she was 5 weeks pregnant. This was the first pregnancy for the patient and there were no risk factors for miscarriage rather than diabetes. Six days after the injection, she was admitted to the hospital due to severe abdominal pain and vaginal bleeding. Miscarriage was diagnosed and she underwent curettage procedure. We concluded that pregnancy tests should be administered prior to intravitreal injection for female patients of reproductive age, and patient testimony should not be the sole reason to dismiss the possibility of pregnancy.

## INTRODUCTION

Intravitreal Anti-vascular endothelial growth factor (Anti-VEGF) agents (IVAs) are frequently used to treat age-related macular degeneration, diabetic retinopathy and retinal vein branch occlusion. Although IVAs are usually used to treat middle-aged and elderly patients, they can be used to treat patients with type 1 diabetes and diabetic macular edema, idiopathic choroid neovascularization and retinal vein branch occlusion sometimes at young ages [1-3]. 

The United States Food and Drug Administration (FDA) classifies Ranibizumab, Bevacizumab and Aflibercept as category C for use during pregnancy, indicating that these drugs have been shown to be toxic to the embryo and fetus in animal studies but evidence in humans is insufficient to draw a conclusion [2]. While the most common systemic side effects of IVAs are hypertension, proteinuria and cerebrovascular attack, they could cause preeclampsia in pregnant patients, lowered weight gain in mothers and fetuses, teratogenic effects on embryos and even miscarriage [3-6]. 

While it is standard practice to obtain patient history related to cardiovascular health for as long as 6 months before any IVA injection, the necessity of assessing the possibility of pregnancy in female patients of reproductive age should not be overlooked. However, instead of relying on patient knowledge, a medical pregnancy test is required to rule out pregnancy. If IVA treatment is deemed necessary, the patient should be warned about the potential risks involved, and, if possible, treatment should be postponed until the first trimester. This case report describes a case of miscarriage which occurred 6 days after an intravitreal Ranibizumab (IVR) injection and underlines the importance of good history taking regarding pregnancy. However, pregnancy tests should be performed prior to intravitreal injection for female patients of reproductive age.

## CASE REPORT

**Figure 1:**
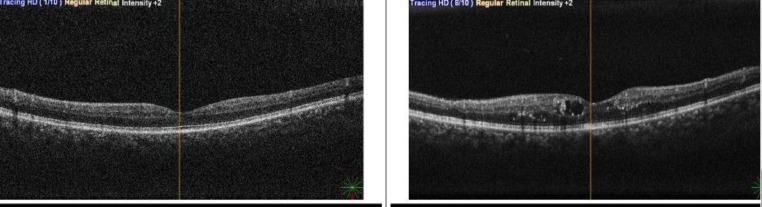
Ocular Coherence Tomography (OCT) Findings Before Intravitreal Ranibizumab Injection Revealed a Cystoid Macular Edema (CME) Around the Fovea and an Increase in Macular Thickness in the Left Eye (Right Figure) and Normal Macular Anatomy and Thickness in the Right Eye (Left Figure).

**Figure 2:**
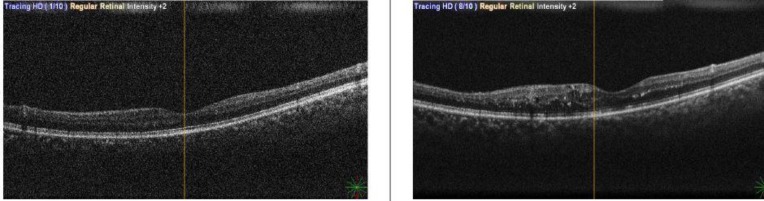
Ocular Coherence Tomography (OCT) Findings One Month After Single Intravitreal Ranibizumab Injection Revealed Decreased Cystoid Macular Edema in the Left Eye (Right Figure) and Normal Macular Anatomy and Thickness in the Right Eye (Left Figure).

The case provided a written, informed consent for the publication of this report. The patient was a 24-year-old female with type 1 diabetes diagnosed at the age of 8. She had been receiving insulin injections 3 times a day, and her Hemoglobin A1C (HbA1c) was in the approximate range of 6–7%. Her last measured HbA1c was 6.4% in the first examination time. She was consulted to ophthalmology clinic of Fatih Sultan Mehmet Training and Research Hospital, Istanbul, Turkey from endocrinology clinic in August 2018. An ophthalmologic examination revealed that the patient’s Snellen corrected distance visual acuity (CDVA) was 10/10 in her right eye and 3/10 in the left eye. Intraocular pressure levels were 12 millimeter of mercury (mmHg) in the right eye and 14 mmHg in the left eye. There were no other complications related to diabetes except for those related to her vision, and her biomicroscopic examination results were normal. A fundus examination revealed diabetic macular edema in the left eye and no noteworthy results in the right eye. Ocular coherence tomography (OCT) further revealed a cystoid macular edema (CME) around the fovea and an increase in macular thickness in the left eye and normal macular anatomy and thickness in the right eye (Fig. 1). The patient was recommended 3 IVR injections at one-month intervals to treat the diabetic macular edema in her left eye. In her pre-injection inquiry, the patient stated that she had been married for 6 months and she was not pregnant. She received 0.05mL/0.5mg IVR in her left eye under sterile condition. During check-up one month later, CME was decreased in her left eye (Fig. 2) but the patient informed us that she had experienced a miscarriage six days after the first injection. She also stated that her period had been late but she was not aware of her pregnancy at the time of intervention. She stated that she had a positive result from a pregnancy test taken two days after the injection. She confirmed the result by visiting an obstetrician and discovered that she was 5 weeks pregnant. This was her first pregnancy and there were no risk factors for miscarriage rather than her diabetes. She had no history of any previous miscarriage, ectopic pregnancy, pelvic surgery or infection. She did not have any related family history and her blood pressure was within the normal range (110/65mmhg) and she had no dyslipidemia. She was admitted to emergency service with severe abdominal pain and vaginal bleeding 6 days after the IVR injection. Miscarriage was diagnosed and a curettage procedure was performed by the obstetrician in charge.

## DISCUSSION

Intravitreal Ranibizumab may cause miscarriage, especially at first trimester. For this reason, we should not rely on a patient history and must take pregnancy test. The ophthalmologist should be careful before intravitreal injection if the patient is female and in the reproductive age. 

Vascular endothelial growth factors (VEGFs) promote vasculogenesis, neoangiogenesis and vascular permeability. Human and animal studies have shown that VEGFs also play an important role in protecting the fetus and placenta, and suppression of plasma VEGF can lead to defects in the embryo and even miscarriage [7]. Intravitreal anti-vascular endothelial growth factor agents are thus unsuitable for use during pregnancy due to systemic side effects they may cause on the mother as well as potential increased risks of harming the fetus, spontaneous miscarriage and preeclampsia [8, 9].

Even though it not yet known whether anti-VEGFs can enter the placenta, it has been shown that these drugs enter the systemic circulation shortly after intravitreal injection [10]. Thus, it would be reasonable to conjecture that anti-VEGFs negatively impact fetal health by entering the systemic circulation and suppressing plasma VEGF levels. Of anti-VEGFs that are currently in use, Ranibizumab clears the fastest from the systemic circulation and has the lowest impact on plasma VEGF levels. Indeed, it was shown that a single IVR injection significantly decreases plasma VEGF levels for just one day. In contrast, a significant decrease in plasma VEGF levels have been observed for at least one month following a single dose of Bevacizumab [11, 12]. There have been three cases reported in the literature of miscarriage following treatment with Bevacizumab and one of these cases had other risk factors for miscarriage. Two of the cases were reported by Petrou et al. aged 29 and 25 who received intravitreal injection during the 4th and 3rd weeks of pregnancy, respectively. The first case had diabetic macular edema known as type 1 diabetes subject, while the second was diagnosed with choroidal neovascularization related to myopia. Miscarriage occurred 7 and 10 days after intravitreal Bevacizumab injections, respectively [13]. The third case was a 41-year-old woman with a miscarriage 7 weeks after a Bevacizumab injection [14]. However, the patient in this report had other risk factors for miscarriage, such as old age. Furthermore, Sullivan et al. reported a patient who developed preeclampsia after 3 doses of intravitreal Bevacizumab, the last of which occurred during pregnancy [6]. Other case reports in the literature describe women who experienced no adverse events during pregnancy despite Bevacizumab injections at similarly early gestational time points [2].

To the best of our knowledge, this is considered as the first report in the literature of a loss of pregnancy after an IVR injection. In this case, the injection was made within the first 5 weeks of pregnancy. In all of the previously described cases of miscarriage following IVA injection, miscarriage occurred 7–10 days after injection. Similarly, miscarriage occurred 6 days after injection in the present case. 

No study has reported any cases of newborn teratogenicity due to treatment with IVAs. However, one case has been published describing premature birth following IVA treatment, although this patient had other risk factors [2]. The timing of IVA treatment during pregnancy is crucial. If IVA treatment is deemed necessary during pregnancy, its benefits and possible harms should be explained to patient in detail. Whenever possible treatment should be postponed until organogenesis is complete at approximately 8-weeks after post-fertilization. 

The risk of spontaneous miscarriage in clinically defined pregnant women has been reported to range from approximately 11% to 16%, and the cases of miscarriage following IVA treatment in the literature may thus be unrelated to exposure to IVAs [15]. However, the common factors described in many of the cases in the literature require attention. Specifically in many of the reported cases including the present case, without any other risk factors, IVR injection within the first 5 weeks of the pregnancy caused miscarriage 6 to 10 days after injection.

## CONCLUSIONS

Intravitreal Ranibizumab may cause miscarriage, especially at first trimester. However, Ranibizumab is the least risky one among other anti-VEGF agents, because Ranibizumab clears the fastest from the systemic circulation and has the lowest impact on plasma VEGF levels.
